# A continuous spectrophotometric assay that distinguishes between phospholipase A_1_ and A_2_ activities[S]

**DOI:** 10.1194/jlr.D065961

**Published:** 2016-08

**Authors:** Meddy El Alaoui, Laurent Soulère, Alexandre Noiriel, Florence Popowycz, Abdallah Khatib, Yves Queneau, Abdelkarim Abousalham

**Affiliations:** * Univ Lyon, Université Lyon 1, UMR 5246, CNRS, INSA Lyon, CPE Lyon, Institut de Chimie et de Biochimie Moléculaires et Supramoléculaires (ICBMS), Métabolismes, Enzymes et Mécanismes Moléculaires (MEM2), F-69622 Villeurbanne, France; † Univ Lyon, INSA Lyon, UMR 5246, CNRS, Université Lyon 1, CPE Lyon, Institut de Chimie et de Biochimie Moléculaires et Supramoléculaires (ICBMS), Chimie Organique et Bioorganique (COB), F-69621 Villeurbanne, France

**Keywords:** tung oil, α-eleostearic acid, β-cyclodextrin, 1-α-eleostearoyl-2-octadecyl-*rac-*glycero-3-phosphocholine, 1-octadecyl-2-α-eleostearoyl-*rac*-glycero-3-phosphocholine

## Abstract

A new spectrophotometric assay was developed to measure, continuously and specifically, phospholipase A_1_ (PLA_1_) or phospholipase A_2_ (PLA_2_) activities using synthetic glycerophosphatidylcholines (PCs) containing α-eleostearic acid, either at the *sn*-1 position [1-α-eleostearoyl-2-octadecyl-*rac*-glycero-3-phosphocholine (EOPC)] or at the *sn*-2 position [1-octadecyl-2-α-eleostearoyl-*rac*-glycero-3-phosphocholine (OEPC)]. The substrates were coated onto the wells of microtiter plates. A nonhydrolyzable ether bond, with a non-UV-absorbing alkyl chain, was introduced at the other *sn* position to prevent acyl chain migration during lipolysis. Upon enzyme action, α-eleostearic acid is liberated and then solubilized into the micellar phase. The PLA_1_ or PLA_2_ activity was measured by the increase in absorbance at 272 nm due to the transition of α-eleostearic acid from the adsorbed to the soluble state. EOPC and OEPC differentiate, with excellent accuracy, between PLA_1_ and PLA_2_ activity. Lecitase^®^, guinea pig pancreatic lipase-related protein 2 (known to be a PLA_1_ enzyme), bee venom PLA_2_, and porcine pancreatic PLA_2_ were all used to validate the assay. Compared with current assays used for continuously measuring PLA_1_ or PLA_2_ activities and/or their inhibitors, the development of this sensitive enzymatic method, using coated PC substrate analogs to natural lipids and based on the UV spectroscopic properties of α-eleostearic acid, is a significant improvement.

Our knowledge of lipolytic enzymes has been substantially improved with the development of analytical technologies and the recognition of their various and important functions in cells. Nevertheless, many mechanisms of lipolytic activity remain unclear and novel technologies are required for further investigations. Phospholipids are major components in the lipid bilayer [50% of the cellular lipids is made up of glycerophosphatidylcholine (PC) (1)], together with plasmatic lipoprotein. Phospholipids can be hydrolyzed by phospholipases A_1_ (PLA_1_s, EC 3.1.1.32) or phospholipases A_2_ (PLA_2_s, EC 3.1.1.4) that catalyze hydrolysis of the ester bond of the acyl group attached to the *sn*-1 or the *sn*-2 position of phospholipid, respectively. At present, the class of PLA_1_ enzymes is not well studied and little structural information is available (2). Some reports have indicated that PLA_1_s could be considered as virulence factors (3) or as being responsible for the production of lysophospholipids that are implicated in many processes, such as angiogenesis or protein transport (4). The endothelial lipase known to be a PLA_1_ is involved in cardiovascular diseases and promoting atherosclerosis, and, consequently, is a major therapeutic target (5). Nevertheless, its mechanism has not been clearly elucidated. Pancreatic lipase-related protein 2 from guinea pigs (GPL-RP_2_) and humans (HPL-RP_2_) has been reported to have triglyceride lipase, PLA_1_, and galactolipase activities (6–8). These enzymes are involved in lipid digestion and their inhibition could reduce obesity, but the physiological involvement of their PLA_1_ activity has not been established (7, 9).

The PLA_2_ superfamily comprises a large group of intracellular (calcium-dependent and -independent) or secreted enzymes, which have been extensively studied with numerous crystal structures [see (10) for review]. Through the production of bioactive fatty acids (e.g., arachidonic acid) and lysophospholipids, PLA_2_s have been implicated in various physiological processes [e.g., inflammation and host defense (11)] and diseases (e.g., asthma, rheumatoid arthritis, and various cancers (12)]. The number of PLA_2_s implicated, and the number of potential inhibitors, is rapidly expanding and this necessitates high throughput specific screening assays in order to discover new potential treatments.

Most of PLA_1_s and PLA_2_s are water soluble lipolytic carboxylester hydrolases capable of releasing long-chain fatty acids from natural water-insoluble carboxylic esters, leading to an enzymatic reaction at the lipid-water interface (13). Consequently, these enzymes do not follow Michaelis-Menten kinetics in which both the enzyme and the substrate must be present in the same phase (13–15). Furthermore, the catalysis reaction strongly depends on the quality of the interface, such as an oil-in-water emulsion, liposomal dispersion, or a monolayer (13, 14), and the use of lipidic substrates containing long-chain fatty acids must be taken into account when accurately assaying the lipolytic activity. All these parameters make it difficult to develop new reliable phospholipase assay systems. Nevertheless, numerous assays using chromogenic (16, 17), radiolabeled (18), or fluorogenic (19–21) substrates, or indirect measurements (22), have been developed over the past decade. Some of them screen phospholipase activities with simple and easy-to-use molecules (16) and others use substrates close to natural lipids (20, 21, 23). However, these labeled substrates often present a sterically hindered fluorochrome group (except with radiolabeled probes) and this may interfere with the lipolytic activity. Phospholipases are lipolytic carboxylester hydrolases capable of releasing long-chain fatty acids from natural water-insoluble carboxylic esters. The use of lipidic substrates containing these long-chain fatty acids must be taken into account when accurately assaying the lipolytic activity.

Crude tung oil (*Aleurites fordii* seed oil) contains up to 70% α-eleostearic acid (9*Z*,11*E*,13*E*-octadecatrienoic acid), a naturally occurring 18-carbon fatty acid esterified mainly at the *sn*-1 and *sn*-3 positions of tung oil triglycerides (24). The conjugated triene present in α-eleostearic acid constitutes an intrinsic chromophore, which confers strong UV absorption properties (24) on both the free fatty acid and the triglycerides. This makes a prime probe with no sterically hindered group. Various lipase assays have been developed based on these properties, using α-eleostearic acid esterified into natural (25, 26) and synthetic triglycerides (27).

With the aim of developing a convenient, specific, sensitive, and continuous UV spectrophotometric assay using a lipidic substrate for monitoring PLA_1_ or PLA_2_ activity, we have recently synthesized a specific PC, named 1,2-α-eleostearoyl-*sn*-glycero-3-phosphocholine (EEPC), esterified at the *sn*-1 and *sn*-2 positions with α-eleostearic acid (28). However, EEPC cannot be used to distinguish between PLA_1_ and PLA_2_ activities due, on the one hand, to the presence of the same fatty acid at the *sn*-1 and *sn*-2 position and, on the other hand, to the migration of the remaining fatty acyl chain yielding lysophosphatidylcholines (lyso-PCs) during lipolysis (29). In this study, we have synthesized, and then used, new PCs containing UV-absorbing α-eleostearic acid at the *sn*-1 [1-α-eleostearoyl-2-octadecyl-*rac*-glycero-3-phosphocholine (EOPC)] or at the *sn*-2 position [1-octadecyl-2-α-eleostearoyl-*rac*-glycero-3-phosphocholine (OEPC)] and a nonabsorbing and nonhydrolyzable *O*-ether alkyl at the other *sn* position, able to continuously monitor the PLA_1_ or PLA_2_ activity, respectively. The design of these new PCs involves the presence of ether bonds, nonhydrolyzable by phospholipases, and, therefore, preventing acyl chain migration during lipolysis, which, in turn, presents a means of discriminating between PLA_1_ and PLA_2_ activity.

## MATERIALS AND METHODS

### Reagents and materials

β-cyclodextrin (β-CD), butylated hydroxytoluene (BHT), DCC (*N*,*N*′-dicyclohexylcarbodiimide), 4-pyrrolidinopyridine (PPyr), Amberlyst 15, and crude tung oil were purchased from Sigma-Aldrich-Fluka Chimie. Methyl indoxam (MI) was kindly provided by Dr. D. Charmot and tetrahydrolipstatin (THL), a known digestive lipase inhibitor, was obtained from Hoffmann-La-Roche Ltd. Microtiter plates (Costar^®^ UV-Plate) were purchased from Corning Inc. TLC aluminum sheets, coated with 0.2 mm silica gel 60 F_254_, were purchased from Merck. All other chemicals and solvents of the highest quality were obtained from local suppliers.

### Proteins

BSA, porcine pancreatic PLA_2_ (ppPLA_2_), honey bee (*Apis mellifera*) venom PLA_2_ (hbPLA_2_), and Lecitase^®^ were all purchased from Sigma-Aldrich-Fluka Chimie. GPL-RP_2_ was kindly provided by Dr F. Carrière. *Candida rugosa* lipase AY30 was obtained from Amano Pharmaceuticals Ltd. The protein concentrations were determined using Bradford’s procedure (30), with Bio-Rad dye reagent and BSA as the standard.

### TLC

Glycerophospholipids were separated by performing analytical TLC on aluminum sheets coated with 0.2 mm silica gel 60. The sample migration was first performed with chloroform/methanol/water (65/35/5, v/v/v), containing 0.001% (w/v) BHT as an antioxidant, until the solvent front was halfway up the plate. The plate was dried and then placed in a second chamber containing hexane/diethyl ether/acetic acid (86/14/1, v/v/v) containing 0.001% (w/v) BHT, until the solvent front reached the top of the plate. The plate was dried again. The various lipids were revealed with UV light at 254 nm (to reveal α-eleostearic-containing species) and by charring the plate after spraying it with 10% copper sulfate and 10% phosphoric acid in water (to reveal all the acyl species).

### Preparation of purified α-eleostearic acid from tung oil

A solution of 20 g of crude tung oil was hydrolyzed with 500 mg of *Candida rugosa* lipase in 14 ml of water, and the reaction was stirred for 3 h at 40°C. Total lipids were extracted into a decantation vial with 100 ml of 3 M HCl and 100 ml of diethyl ether containing 0.01% BHT (w/v). The organic layer was recovered, dried by adding anhydrous MgSO_4_, filtered, and concentrated under reduced pressure. The total lipid extract (5 g), containing mainly free fatty acids, was further purified by recrystallization in 5.5 ml of acetone, at 60°C for 20 min, and then cooled on ice. The heterogeneous mixture was filtered and the crystalline solid obtained was treated with dry acetone. The crystals were then collected by filtration and dried in vacuo (2.2 g, 40% yield from the lipolysis extract).

### Synthesis of EOPC and OEPC

See the supplemental information for details.

### Coating microtiter plates with synthetic phospholipids

Microtiter plates were coated with EOPC or OEPC, as described previously (26–28). The phospholipid solution (0.5 mg·ml^−1^) was prepared in ethanol, containing 0.001% BHT as an antioxidant, and the wells of the UV-microtiter plate were filled with phospholipids (100 μl/well). The microtiter plate was first partially dried under a fume hood and then left in a vacuum desiccator until the solvent had completely evaporated (around 30 min). After ethanol evaporation, the coated EOPC or OEPC plates were found to be stable in the dark for at least 1 week at 4°C.

### UV spectrophotometric PLA_1_ and PLA_2_ assays using coated synthetic phospholipids

The PLA_1_ and PLA_2_ activities were assayed spectrophotometrically by measuring the amount of α-eleostearic acid continuously released from the phospholipid substrates. The enzyme activity measurement was performed using 10 mM Tris-HCl buffer (pH 8.0) containing 3 g·l^−1^ β-CD, 150 mM NaCl, 6 mM CaCl_2_, and 1 mM EDTA. The nontensioactive β-CD was used in the reaction buffer in order to solubilize the long-chain lipolytic products. The substrate was dissolved in ethanol to obtain the desired final concentration and the wells of a 96-well flat-bottomed microtiter plate were then coated with the lipids, as described above. The substrate-coated wells were subsequently washed with the assay buffer and left to equilibrate for 10 min at 37°C. The assays were run in a 200 μl final volume at 37°C. The enzyme solutions (2–10 μl) were injected into the microtiter plate wells and the absorbance at 272 nm was recorded continuously at regular 1 min intervals against the buffer alone. A microtiter plate-scanning spectrophotometer (Tecan Infinite M200 Pro) was used. The plate was shaken for 5 s before each reading.

The specific activity of various enzymes used in this work was calculated from the steady-state reaction rate, expressed as the change in absorbance per minute using an apparent molar extinction of 5,320 M^−1^ · cm^−1^ (see the Results and Discussion). The PLA_1_- or PLA_2_-specific activity was expressed as international units per milligram of enzyme. One international unit corresponds to 1 μmol of fatty acid released per minute under the assay conditions.

Alternatively, after various periods of time, the hydrolytic reaction was stopped by acidification, using 20 μl of 0.1 M HCl, and the total aqueous phase (220 μl) was transferred to a glass vial. In order to maximize total lipid recovery, each well was washed twice with 100 μl of ethanol and added to the aqueous phase. Lipids were then extracted with 1 ml of chloroform/methanol (2/1, v/v), shaken vigorously, and centrifuged for 1 min at 1,000 *g*. The lower organic phase was collected and transferred to a new glass tube and dried with anhydrous MgSO_4_. MgSO_4_ was removed by centrifugation for 1 min at 1,000 *g* and the clear lipid extract was transferred to a 2 ml vial. The remaining solvent was evaporated, under nitrogen flow to prevent oxidation, and the lipids were dissolved in 20 μl of a mixture of chloroform/methanol (2/1, v/v) containing 0.001% BHT. A TLC analysis of the lipids was carried out using TLC precoated plates 60 F_254_, as described above.

### Microtiter plate assay to study PLA_1_ and PLA_2_ inhibition

The enzyme-inhibitor incubation method (31) was used to test, in aqueous medium and in the absence of the substrate, whether any direct interactions could occur between the enzyme and the inhibitor. GPL-RP_2_ (0.5 μg, 69 nM) was incubated at 25°C with THL solution (4.8 μM in DMSO) for 1 h at an enzyme:inhibitor molar ratio of 1:70 (final DMSO concentration 2.5%). ppPLA_2_ (0.5 μg, 140 nM) was incubated at 25°C with MI solution (14 μM in DMSO) for 1 h at an enzyme:inhibitor molar ratio of 1:100 (final DMSO concentration 2.5%). The residual enzyme activities were then measured using either EOPC or OEPC coated onto the surface of the microtiter plate wells.

### Statistical analysis

Data are expressed as mean ± SD. Statistical significance was determined by the Student’s unpaired *t*-test (two-tailed). Samples were considered to be significantly different for *P* < 0.01 (**).

## RESULTS AND DISCUSSION

**Fig. 1.**
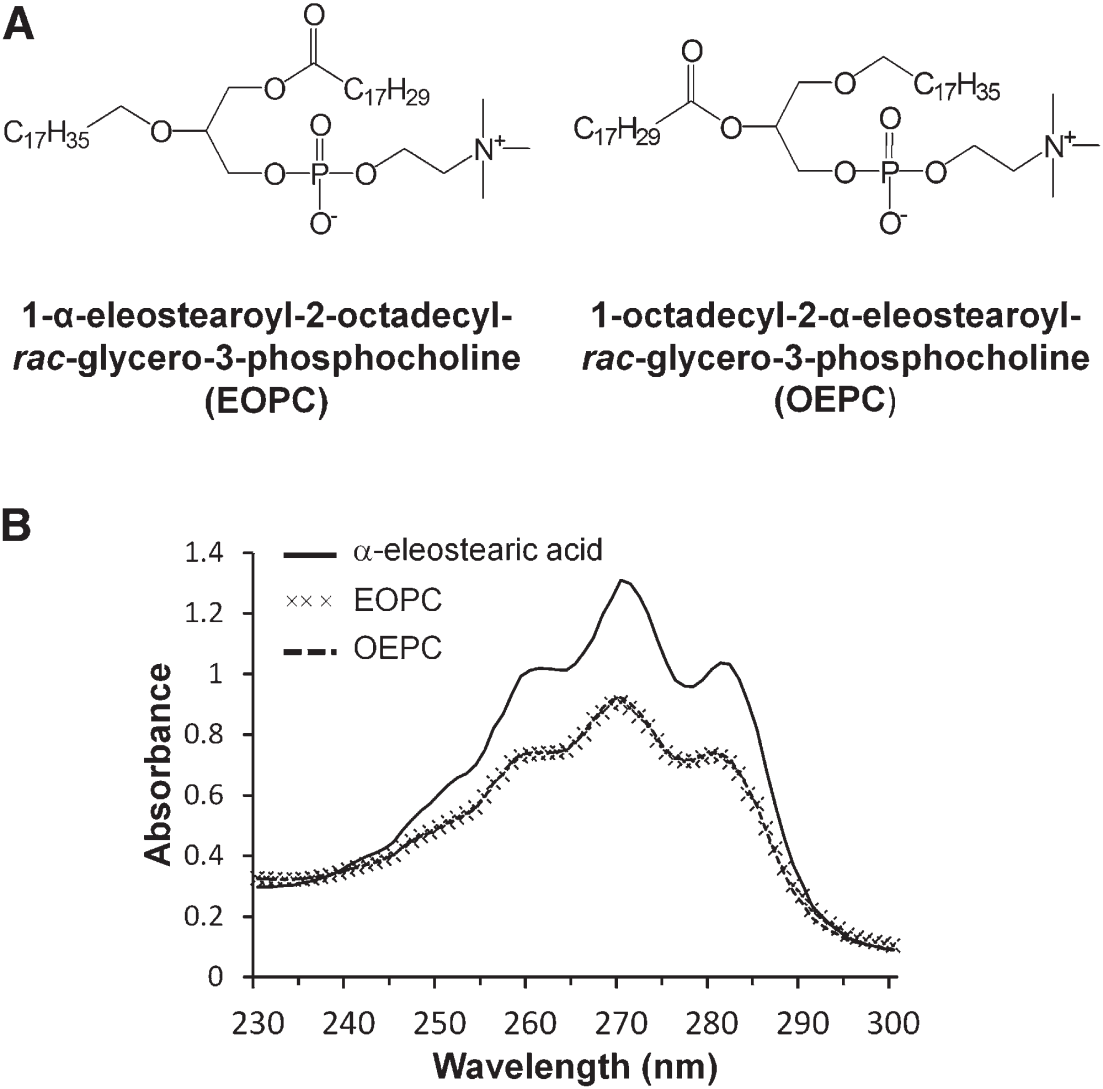
Chemical structure and UV absorption spectra of EOPC and OEPC. A: Chemical structure of EOPC and OEPC. B: UV absorption spectra of α-eleostearic acid (62.5 μg ml^−1^), EOPC (125 μg ml^−1^) and OEPC (125 μg ml^−1^) dissolved in ethanol containing BHT 0.001%.

In our previous published work (28), we synthesized a specific PC (EEPC), esterified at both *sn*-1 and *sn*-2 positions with α-eleostearic acid and coated onto microtiter plate wells to monitor the PLA_1_ or PLA_2_ activities continuously. However, the fact that the EEPC substrate was esterified at both *sn* positions of the PC with α-eleostearic acid made it impossible to distinguish between the activities of these two enzymes. In the present work, two structural analogs of PC esterified with α-eleostearic acid, either at the *sn*-1 (EOPC) or at the *sn*-2 (OEPC) position, and containing a non-UV-absorbing and nonhydrolyzable ether bond at the second position (**Fig. 1A**), were synthesized and used as substrates to discriminate between PLA_1_ and PLA_2_ activities. The ether bond was introduced to prevent intramolecular acyl group migration during lipolysis by PLA_1_ or PLA_2_.

### Synthesis of EOPC and OEPC, and their spectroscopic properties

The α-eleostearic acid was obtained from tung oil enzymatic hydrolysis, and it was recrystallized from the total lipid extract. The specific UV spectrum obtained showed three majors peaks located at λ max (ethanol 95%) = 260, 270, and 280 nm (Fig. 1B). The synthesis of EOPC and OEPC (supplemental Fig. S1) was achieved in eight steps from *rac*-glycidol, as summarized in the supplemental information. Because the usual coupling agent, DCC with PPyr (32), for the final acylation (supplemental Fig. S1, compounds 8 and 15) gave poor results, another method was tested using oxalyl chloride (33) to generate the acyl chloride of α-eleostearic acid before the final reaction, but without success. However, better results were observed with DCC/PPyr, as used by Borsotti et al. (34), promoting the acylation of lyso-PC (supplemental Fig. S1, compounds 7 and 14) with anhydride fatty acid derivatives, giving acceptable yields of 48 and 25% for EOPC and OEPC, respectively. The difference in the yields between the two substrates may be due to a steric hindrance at position *sn*-2. Moreover, the rather low yield obtained for this last step could be due to the specific treatments applied to eliminate any ionic species by exchange ionic chromatography (similar amphiphilic properties of PCs and PPyr) during the purification process.

The final yields for the total synthesis of EOPC and OEPC were measured at 12 and 7%, respectively, over the eight steps. The ^1^H NMR spectrum of EOPC (supplemental Fig. S2A) and OEPC (supplemental Fig. S2B) showed signals of the vinylic system, between δ 6.4 and 5.6 parts per million (ppm), as a complex set of multiplets. A single signal at δ 5.4 ppm is characteristic of the *sn*-2 single proton. At δ 4.4 ppm to δ 3.7 ppm, multiplets are referenced to protons close to the glycerol chain group (*sn*-1 and *sn*-3 positions and the phosphatidyl group). The methyl groups of the quaternary ammonium gave a specific signal at δ 3.4 ppm. Saturated protons from the carbonyl chain are located from δ 2.3 ppm (α carbon) to δ 0.9 ppm (ω carbon).

As shown in Fig. 1B, the UV absorption spectrum (230–300 nm) of an ethanolic solution of EOPC and OEPC displayed three major peaks located at 260, 270, and 280 nm. This profile spectrum is similar to that of pure α-eleostearic acid [Fig. 1B and (26)], pure tung oil triglycerides (25), synthetic triglycerides (27), and PC-containing α-eleostearic acid (28). In aqueous buffer, a solvatochromic effect induced a 2 nm redshift of the UV absorption spectrum in which the major absorption peak was shifted from 270 to 272 nm, as described earlier (35).

### Principle of the PLA_1_ and PLA_2_ assay

**Fig. 2.**
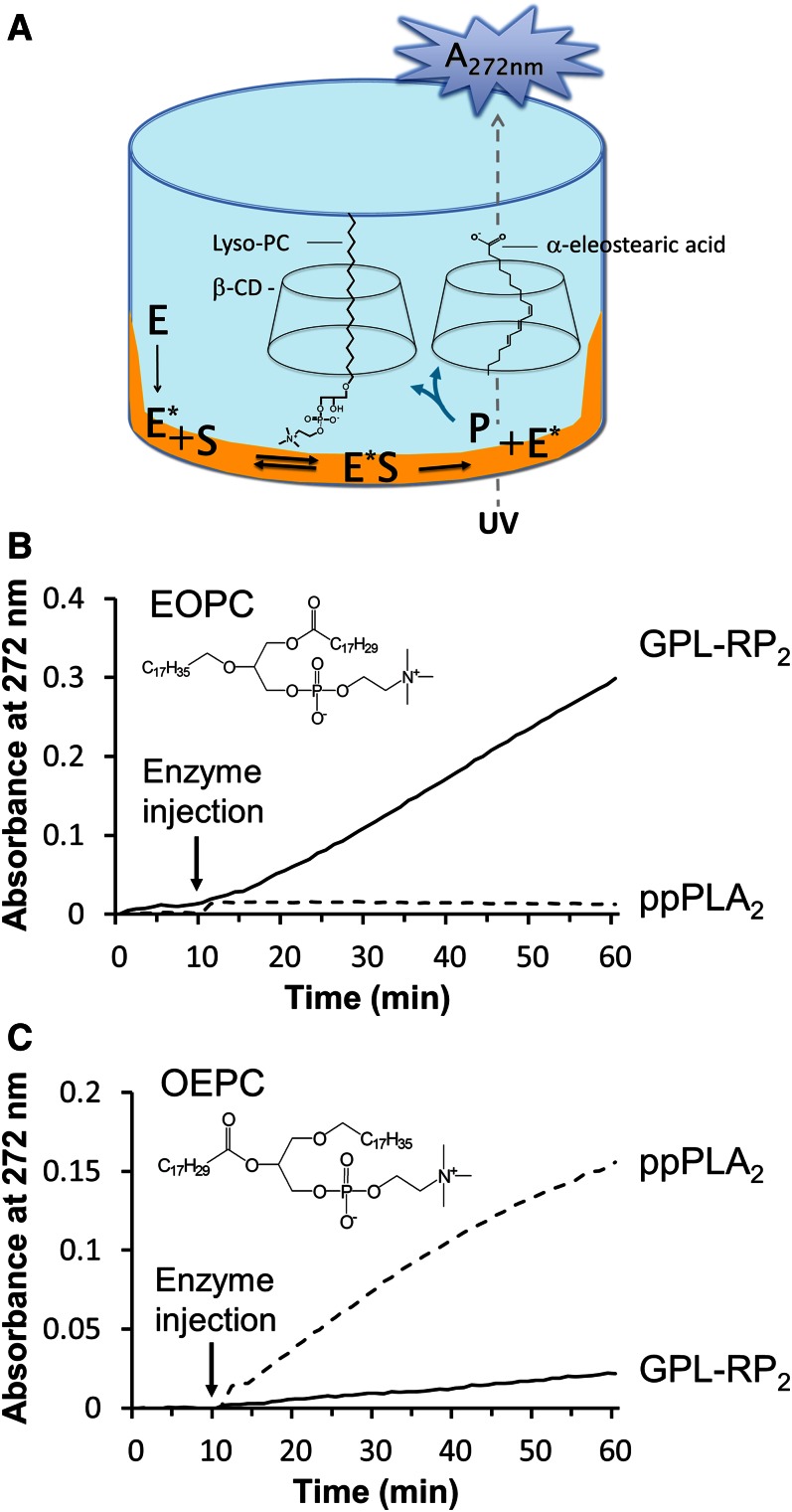
Principle of the coated PC assay and time course of the enzymatic hydrolysis of OEPC or EOPC coated on microtiter plate wells. A: Schematic representation of the assay reaction showing the hydrolysis of coated EOPC or OEPC in a microwell. E, enzyme in solution; E*, enzyme at the interface; S, substrate (EOPC or OEPC); P, lipolysis products (α-eleostearic acid and non-UV-absorbing lyso-PC). β-CD (3 g·l^−1^, final concentration) was used to solubilize the long-chain lipolysis products into the aqueous buffer. B, C: Kinetic recordings of coated EOPC (B) and OEPC (A) lipolysis by GPL-RP_2_ or ppPLA_2_. Variations, with time, of the absorbance at 272 nm were recorded for 10 min, for stabilization, and then for 50 min after GPL-RP_2_ or ppPLA_2_ (0.5 μg per well). The kinetic recordings shown here are typical of those obtained in three independent experiments.

The principle of the assay using coated PCs has been described in our previous report (28) and is schematically shown in **Fig. 2A**. PCs (EOPC or OEPC) were first dissolved in ethanol containing BHT (0.001%, w/v) and injected into microtiter plate wells. The UV absorption spectrum obtained was characteristic of pure α-eleostearic acid, with three major peaks located at 260, 270, and 280 nm, respectively. After ethanol evaporation under vacuum, the PCs remained as a thin film coating the wells. The assay buffer containing the nontensioactive β-CD was then added and the thin PC films exhibited a UV spectrum with very low absorbance, which could not be desorbed by any interaction with the aqueous buffer (28). Once injected in the microtiter plate well, the enzyme (PLA_1_ or PLA_2_) in solution (E, enzyme in solution, Fig. 2A) can bind to the interface (E*, enzyme at the interface, Fig. 2A), where the hydrolysis of coated PCs is performed by the release of lipolysis products. The long-chain lipolysis products (α-eleostearic acid and lyso-PC) were solubilized into the aqueous buffer containing β-CD. β-CD (seven glucopyranoside units) was reported to form inclusion complexes with free fatty acids, as well as with monoglycerides (36) and lyso-PC (15, 37). The desorption rate of the water-insoluble reaction products (α-eleostearic acid and non-UV-absorbing lyso-PC) probably involves the complexation of the single acyl chain into the β-CD hydrophobic cavity and the desorption into the aqueous phase of either soluble α-eleostearic acid/β-CD or non-UV-absorbing lyso-PC/β-CD complexes. The solubilization of the long-chain lipolysis products prevents their accumulation at the interface, which can lead to a modification of the “interfacial quality” of the lipid structures (14). Moreover, the UV absorbance of α-eleostearic acid was considerably enhanced due to its transition from the adsorbed state to the soluble state, and the lipolytic enzyme activity could be followed continuously by recording the variations of the UV absorption spectra with time. The optimal final concentration of β-CD in the reaction buffer has been previously optimized and shown to be 3 g·l^−1^ (28).

### Enzyme kinetic recordings using coated EOPC and OEPC as substrates

To validate the assay method, hydrolysis of coated EOPC and OEPC was performed using GPL-RP_2_, Lecitase^®^, ppPLA_2_, and hbPLA_2_. GPL-RP_2_ belongs to the pancreatic lipase gene family and, in contrast to classical guinea pig pancreatic lipase, it has the ability to hydrolyze PC at the *sn*-1 position (9, 38). Lecitase^®^ is a commercially available enzyme obtained from the fusion of the genes of *Thermomyces lanuginosus* lipase and *Fusarium oxysporum* phospholipase, and known to hydrolyze the *sn*-1 position of PC specifically (39). As shown in Fig. 2B, C, a constant baseline was recorded prior to enzyme injection, indicating that the various PCs were not desorbed by any interaction with the buffer components. The same results were obtained when injecting heat-inactivated enzymes, indicating that PCs were also not desorbed by any interaction with proteins (data not shown).

Following an injection of GPL-RP_2_ (Fig. 2B) or Lecitase^®^ (data not shown) onto coated EOPC, the absorbance at 272 nm increased rapidly, confirming the high PLA_1_ activity of these lipases. However, no hydrolysis was observed when injecting either ppPLA_2_ (Fig. 2B) or hbPLA_2_ (data not shown) onto coated EOPC due to the presence of a nonhydrolyzable ether bond at the *sn*-2 position of the PC substrate.

Following the addition of ppPLA_2_ (Fig. 2C) or hbPLA_2_ (data not shown) to coated OEPC, the reaction rate increased in a time-dependent manner, as reflected by the increase of absorbance measured at 272 nm. Interestingly, the addition of GPL-RP_2_ (Fig. 2C) or Lecitase^®^ (data not shown) onto coated OEPC showed a weak, but significant, linear increase of absorbance at 272 nm. These findings indicate that GPL-RP_2_ is able to release free α-eleostearic acid from the *sn*-1 position of EOPC, and that Lecitase^®^ is able to release it from the *sn*-2 position of OEPC. However, the PLA_2_ activity measured for GPL-RP_2_ and Lecitase^®^, using OEPC, was found to be around 10- and 28-fold lower, respectively, than their PLA_1_ activity using EOPC (see Table 1).

The PLA_1_ activity previously reported (6–8) for pancreatic lipase-related protein 2 has usually been measured potentiometrically and continuously using egg PC as the substrate. However, this assay is not specific to the hydrolysis of the ester bond at the *sn*-1 position of the PC substrate and, consequently, it does not take into account a possible PLA_2_ activity. The ability of pancreatic lipases-RP_2_ to hydrolyze both the ester bonds at the *sn*-1 and *sn*-2 positions of PC has been previously reported (40) for HPL-RP_2_, using 1-palmitoyl-2-[1-^14^C] arachidonyl-*sn*-glycero-3-phosphocholine as the substrate. HPL-RP_2_ was found to hydrolyze both the ester bonds at the *sn*-1 and *sn*-2 positions of the PC substrate and the PLA_1_ activity was estimated to be almost two times higher than the PLA_2_ activity of HPL-RP_2_, as revealed by the radioactivity measured in the lipolysis product bands from TLC chromatogram (40). The radiometric assay is, however, tedious and discontinuous and could not be used for high throughput screening. GPL-RP_2_ and HPL-RP_2_ belong to the pancreatic lipase gene family and, compared with classical lipases, they are reported to be more hydrophilic. Thus, GPL-RP_2_ and HPL-RP_2_ are able to accommodate phospholipids and galactolipids with large polar heads (7).

### TLC analysis of the lipolysis products

**Fig. 3.**
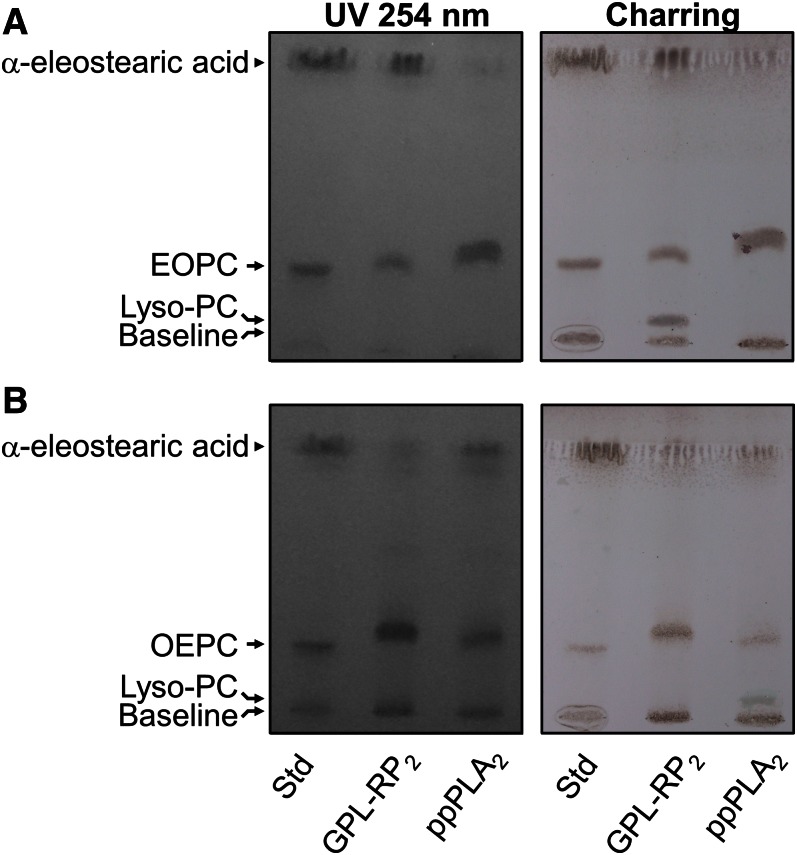
Analysis by TLC of coated EOPC (A) and OEPC (B) hydrolysis revealed with UV light at 254 nm (left panels) and with 10% copper sulfate and 10% phosphoric acid in water followed by charring at 150°C for 15 min (right-hand panels). The standards (Std) used (10 μg of each compound) were α-eleostearic acid, EOPC, and OEPC. Coated PCs were hydrolyzed by GPL-RP_2_ or ppPLA_2_ (0.5 μg/well). The chromatographic solvent system was chloroform/methanol/water (65/35/4, v/v/v) and hexane/diethylether/acetic acid (84/16/1, v/v/v) containing 0.001% (w/v) BHT.

To further validate the assay method, the lipolysis products were extracted from the wells of the microtiter plates and analyzed using TLC. The TLC analysis of the lipolysis products of GPLR-RP_2_ on EOPC showed a qualitative decrease in EOPC content and the appearance of α-eleostearic acid, but not nonabsorbing lyso-PC, as revealed with UV light (**Fig. 3A**, UV 254 nm). When a TLC plate from the same experiment with EOPC was analyzed by charring, the appearance of lyso-PC, in addition to α-eleostearic acid, was clearly observed (Fig. 3A, charring). In contrast to GPL-RP_2_, and as expected, ppPLA_2_ showed no hydrolysis products using EOPC as the substrate, as revealed with UV 254 nm and charring (Fig. 3A).

The TLC analysis of the lipolysis products of a ppPLA_2_-catalyzed hydrolysis of OEPC clearly showed a qualitative decrease in the OEPC content and the appearance of UV absorbing α-eleostearic acid (Fig. 3B, UV 254 nm) and non-UV-absorbing lyso-PC (Fig. 3B, charring). It is worth noting that the production of lyso-PC, due to the action of GPL-RP_2_ on EOPC (Fig. 3A, charring) or ppPLA_2_ on OEPC (Fig. 3B, charring), is only visible by lipid charring because of the absence of UV-absorbing groups (α-eleostearic acid) in octadecyl-PC. The TLC analysis of the lipolysis products of a GPL-RP_2_-catalyzed hydrolysis of OEPC showed no significant decrease in the initial substrate content (Fig. 3B). This result is in contrast with the observed PLA_2_ activity of GPL-RP_2_ using OEPC as the substrate, shown in Fig. 2C. As indicated earlier, the PLA_2_ activity of GPL-RP_2_ on OEPC was seen to be almost 10 times lower than its activity on EOPC under the same experimental conditions (see **Table 1**). Consequently, TLC is not sensitive enough to reveal the lipolysis products of a GPL-RP_2_-catalyzed hydrolysis of OEPC.

**TABLE 1.** Estimation of enzyme specific activities using EOPC or OEPC coated on microtiter plate wells

	Specific Activity (μmol·min^−1^·mg^−1^)			
Enzyme/Substrate	Lecitase^®^	GPL-RP_2_	ppPLA_2_	hbPLA_2_
EOPC	2.20 ± 0.30	0.300 ± 0.019	0*^a^*	0*^a^*
OEPC	0.08 ± 0.01	0.030 ± 0.003	0.250 ± 0.002	0.780 ± 0.039
EOPC/OEPC ratio	27.5	10	0	0

Comparison of specific activities of Lecitase^®^, GPL-RP_2_, ppPLA_2_, and hbPLA_2_ using EOPC or OEPC coated on microtiter plate wells. Results are given as mean ± SD for three independent experiments.

a Below the detection limit, estimated as three times the background obtained with buffer alone.

### Influence of the initial PC concentration and the amount of enzyme on the steady-state reaction rates

**Fig. 4.**
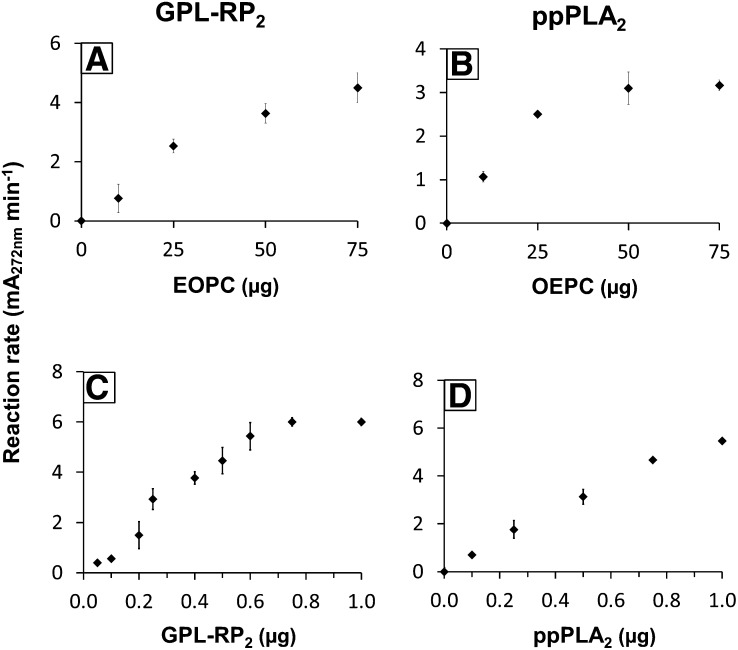
Effects of the initial PC concentration (A, B) and the amount of enzyme (C, D) on the steady-state reaction rates. Variable amounts of coated EOPC (A) or OEPC (B) were subjected to hydrolysis by GPL-RP_2_ [0.5 μg (A)] or ppPLA_2_ [0.5 μg (B)]. Variable amounts of GPL-RP_2_ (C) or ppPLA_2_ (D) were injected into the microplate well containing the coated EOPC (C) or OEPC (D) in 200 μl of standard buffer. The increase in the absorbance at 272 nm was recorded for 20–40 min after the enzyme injection, and the initial velocity (ΔmA_272nm_·min^−1^) was used for reaction rate determination. Results are given as mean ± SD for three independent experiments.

Coated microtiter plates were prepared with variable amounts of OEPC or EOPC ranging from 0 to 75 μg per well and their lipolysis by GPL-RP_2_ (**Fig. 4A**) or ppPLA_2_ (Fig. 4B) was performed. The steady state reaction rate (slope of the variations, with time, in the absorbance at 272 nm) were plotted as a function of the amounts of coated EOPC (Fig. 4A) or OEPC (Fig. 4B). The lipolysis reaction rates increased quasi-linearly with the amounts of PCs, up to 25 μg per well, and then decreased slightly with higher PC amounts (Fig. 4A, B). Based on these results, a final PC amount of 50 μg per well was selected for further kinetic experiments. The same amount of coated substrate per well was previously used and reported for the lipolysis of coated tung oil triglycerides (26), as well as coated synthetic triglycerides (27) or PC-containing (28) α-eleostearic acid.

Using coated EOPC (50 μg per well) as the substrate, the steady-state reaction rate was found to be linear from 0.2 to 0.6 μg/well for GPL-RP_2_ (Fig. 4C) and from 0 to 0.15 μg/well for Lecitase^®^ (supplemental Fig. S3A). At a higher concentration of enzyme, any increase in the reaction rate was probably due to an excess of enzyme. It was established that as little as 10 ng of GPL-RP_2_ and 1 ng of Lecitase^®^ can be detected under standard conditions. These measurements correspond to three times the background observed with buffer alone. Similarly, when using coated OEPC, the increase, with time, of the absorbance at 272 nm was recorded after injecting variable amounts of ppPLA_2_ (Fig. 4D) or hbPLA_2_ (supplemental Fig. S3B). In both cases, the steady-state reaction rate was found to be linear with time and proportional to the range of 0–0.8 μg of enzyme per well. The detection limit, under standard conditions, was as low as 5 ng of ppPLA_2_ and hbPLA_2_. A similar sensitivity of the assay toward these enzymes has been previously observed using coated EEPC as the substrate (28). This assay shows a 100–1,000 times higher sensitivity of enzyme detection than the pH-stat method (1 μg), which is routinely used to measure PLA_2_ activity with PC (41). Moreover, the small amount of PLA_2_ (5 ng) detected in our assay system was shown to be in the same range as that reported for PLA_2_ from *Naja naja* (42) (1 ng) and for calcium-dependent secretory PLA_2_-IIA (43) (0.4 ng), using synthetic fluorescent substrates. Nevertheless, this method is still less sensitive than the radiometric or gold standard mass spectrometry end point methods (44, 45).

### Estimation of the specific activity

The apparent molar extinction coefficient (ε_app_) of α-eleostearic acid has been previously determined by recording the absorbance, at 272 nm, of coated EEPC and various amounts of α-eleostearic acid per well, and it was found to be 5,320 ± 160 M^−1^ · cm^−1^ (28). Under these conditions, the increase, with time, of the absorbance at 272 nm (A_272 nm_ · min^−1^) was converted into μmol · min^−1^ · mg^−1^ of enzyme.

Using coated EOPC as the substrate, the specific activity of Lecitase^®^ and GPL-RP_2_ was calculated to be 2.2 and 0.3 μmol · min^−1^ · mg^−1^, respectively (Table 1). As expected for coated EOPC with an ether bond at the *sn*-2 position, no detectable activity was found for ppPLA_2_ (Fig. 2B, Table 1) or hbPLA_2_ (Table 1). For the sake of comparison, the specific activity of pure GPL-RP_2_ on egg yolk PC, using the pH-technique, was shown to be 45 μmol · min^−1^ · mg^−1^ (46). It is important to note that the stirring and emulsification conditions differ considerably depending on whether a microplate or a mechanically stirred pH-stat vessel is used. Using coated OEPC as the substrate, the specific activities of ppPLA_2_ and hbPLA_2_ were found to be 0.25 and 0.78 μmol · min^−1^ · mg^−1^, respectively (Table 1). Using PC analog with a BODIPY-labeled alkyl ether at the *sn*-1 position and a *N*-(DNP)-8-amino-octanoyl group at the *sn*-2 position as substrate, Hendrickson et al. (20) showed a specific activity of around 18 nmol · min^−1^ · mg^−1^ for cytosolic (85 kDa) PLA_2_.

Under the same experimental conditions, the specific activity of Lecitase^®^ and GPL-RP_2_ was calculated to be 0.08 and 0.03 μmol · min^−1^ · mg^−1^, respectively (Table 1). The activity of Lecitase^®^ and GPL-RP_2_ on EOPC was found to be approximately 28 times and 10 times higher than on OEPC, respectively (Table 1). Access to the active site of classical pancreatic lipase is controlled by a surface loop, the lid, which normally only undergoes conformational changes following the addition of lipids or amphiphiles. A deletion within the lid domain was, however, observed in GPL-RP_2_, which is able to accommodate more hydrophilic substrates, such as phospholipids and galactolipids, with large polar head groups (47), than classical pancreatic lipase. The addition of the classical pancreatic lipase lid to GPL-RP_2_ has been shown to decrease, but not abolish, the phospholipase activity, suggesting that the lid contributes to substrate specificity (48). Moreover, GPL-RP_2_ was shown to be produced at a high level in guinea pigs lacking pancreatic PLA_2_, suggesting a significant role of this enzyme in phospholipid digestion (49). Our study demonstrates for the first time, to the best of our knowledge, a PLA_2_ activity of GPL-RP_2_ and Lecitase^®^ in addition to their triglyceride lipase and PLA_1_ activities.

### Application of the microplate assay for screening potential PLA_1_ or PLA_2_ inhibitors

An important feature of this work is the ability to screen new potential PLA_1_- or PLA_2_-specific inhibitors. THL and MI are known to inhibit digestive lipases (7, 50) and PLA_2_ (51), respectively.

**Fig. 5.**
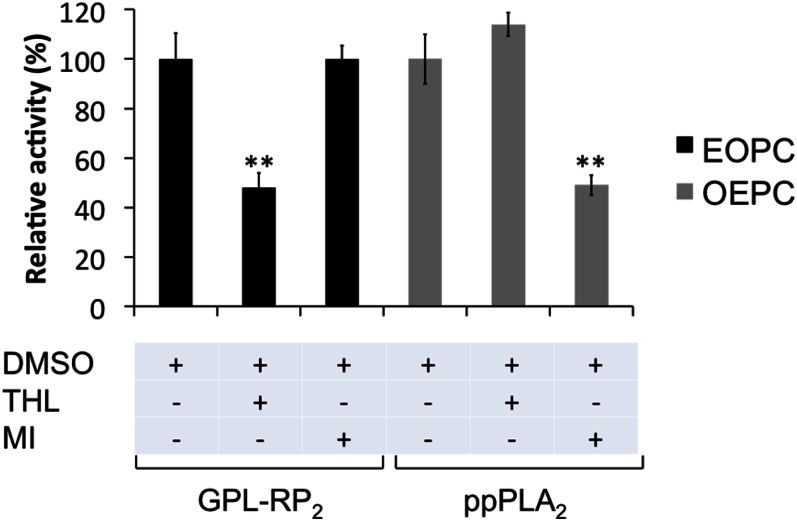
Validation of the assay to study PLA_1_ or PLA_2_ inhibition. Either GPL-RP_2_ or ppPLA_2_ was incubated with THL (enzyme:inhibitor, molar ratio of 1:70) or with MI (enzyme:inhibitor, molar ratio of 1:100) in the absence of the substrate. The residual activities of GPL-RP_2_ or ppPLA_2_ were then measured using coated EOPC or OEPC, respectively, as described in the Materials and Methods, and expressed as a percentage relative to the activity measured in the absence of inhibitor. Values ± SD are the mean of three independent experiments. **P < 0.01 (vs. DMSO).

Using coated EOPC as the substrate, GPL-RP_2_ was inhibited by about 50% when preincubated for 1 h at an enzyme:THL molar ratio of 1:70 (**Fig. 5**). By contrast, preincubating MI with GPL-RP_2_ does not affect the catalytic activity of this enzyme measured under the same experimental conditions (Fig. 5). Similarly, preincubating THL with Lecitase^®^ for 1 h at a molar excess of 100 leads to 40% inactivation of the lipolytic activity observed with coated EOPC (data not shown). THL is known to be a digestive lipase catalytic inhibitor and it has been shown to inhibit the triglyceride lipase activity of GPL-RP_2_, even in the absence of an interface (7). The fact that both the lipase and phospholipase activities of GPL-RP_2_ and Lecitase^®^ were inhibited by THL is an indication that the same catalytic environment is probably involved for triglyceride lipase, PLA_1_, and PLA_2_ activities of these lipolytic enzymes.

Using coated OEPC as the substrate, an injection of ppPLA_2_, preincubated (for 1 h) with a 100 molar excess of MI, leads to approximately 50% inactivation of the enzyme (Fig. 5). As expected (52) and under the same experimental conditions, ppPLA_2_ was not inhibited by THL (Fig. 5). In our previous report (28), we have shown that the preincubation of ppPLA_2_ (1 h) with a 50 or 100 molar excess of MI reduced the enzyme activity by 69% or 79%, respectively.

## CONCLUSIONS

In the method described in this work, new synthetic PC derivatives with close structural analogy to natural substrates were designed, esterified with a naturally occurring conjugated polyene fatty acid with specific optical properties, and substituted with one non-UV-absorbing ether-bonded chain. Because these ether bonds are nonhydrolyzable by PLA_1_ or PLA_2_, no acyl chain migration could occur during lipolysis, thus providing the possibility of discriminating accurately between PLA_1_ and PLA_2_ activities. In addition, the lipidic character of these PC analogs presents a significant qualitative advantage over other methods. Furthermore, this assay is specific, continuous, and sensitive, and it allows screening of new PLA_1_ and PLA_2_ activities and/or their inhibitors present in various biological samples. Using this new enzymatic assay, we have demonstrated for the first time, to the best of our knowledge, the existence of the PLA_2_ activity of GPL-RP_2_ and Lecitase^®^ in addition to their triglyceride lipase and PLA_1_ activities.

## Supplementary Material

Supplemental Data
