# Supplementary material for: A continuous spectrophotometric assay that distinguishes between phospholipase A1 and A2 activities
Source: J Lipid Res. 2016 Aug;57(8):1589–97. doi: 10.1194/jlr.D065961 (PMC4959851; doi:10.1194/jlr.D065961)
Supplement: Supplemental Data [file supp_57_8_1589__index.html]

A continuous spectrophotometric assay that distinguishes between phospholipase A1 and A2 activities — A continuous spectrophotometric assay that distinguishes between phospholipase A1 and A2 activities — Supplemental Data 

# A continuous spectrophotometric assay that distinguishes between phospholipase A1 and A2 activities

## Supplemental Data

- Supporting information (.pdf, 569 KB) - Supporting information
