# Supplementary material for: A continuous spectrophotometric assay that distinguishes between phospholipase A1 and A2 activities
Source: J Lipid Res. 2016 Aug;57(8):1589–97. doi: 10.1194/jlr.D065961 (PMC4959851; doi:10.1194/jlr.D065961)
Supplement: Supplemental Data [file 10.1194_D065961_jlr.D065961-1.pdf]

A continuous spectrophotometric assay that distinguishes between phospholipase A_1 and A_2 activities

Meddy El Alaoui[†,‡], Laurent Soulère[‡], Alexandre Noiriel[†], Florence Popowycz[‡],Abdallah Khatib[†], Yves Queneau[‡] and Abdelkarim Abousalham[†*]

[†] Univ Lyon, Université Lyon 1, Institut de Chimie et de Biochimie Moléculaires et Supramoléculaires, UMR 5246, Métabolisme, Enzymes et Mécanismes Moléculaires (MEM2), 43, Bd du 11 novembre 1918, F-69622 Villeurbanne cedex, France.

[‡] Univ Lyon, Université Lyon 1, INSA Lyon, CPE Lyon, Institut de Chimie et de Biochimie Moléculaires et Supramoléculaires, UMR 5246, Chimie Organique et Bioorganique, Bâtiment Jules Verne, 20 Avenue Albert Einstein, 69621 Villeurbanne Cedex, France.

SUPPORTING INFORMATION

Synthesis of phosphatidylcholines containing α-eleostearic acid: 1-α-eleostearoyl-2-octadecyl-*rac*-glycero-3-phosphocholine (EOPC) and 1-octadecyl-2-α-eleostearoyl-*rac*-glycero-3-phosphocholine (OEPC)

Synthesis of EOPC

Trityl glycidol (1)
The synthesis of trityl glycidol (**1**) was achieved from *rac*-glycidol, according to literature (1)

1-Benzyl-3-trityl-*rac*-glycidol (2)
According to literature (2), to a stirred solution of NaH (60% dispersion in oil, 1.27 g, 34 mmol) in DMF (12 mL), a solution of benzyl alcohol (3.2 mL, 34 mmol) in DMF (6 mL) was stirred at room temperature for 15 min. Then a solution of trityl glycidol (1) (**1**; 2 g, 6.3 mmol) in DMF (5 mL) was added dropwise and the mixture was stirred at 100°C for 2.5 h. After cooling, the mixture was diluted in H_2O (20 mL) and extracted with ether (3 x 30 mL). The combined organic layer was dried with $MgSO_4$ and evaporated. Excess of benzyl alcohol (BnOH) was distillated off (bath temperature 100°C, 130 Pa) and the residue was purified by column chromatography on silica gel (120 g, Ethyl acetate/petroleum ether, 25:75, v/v) to afford 1-benzyl-3-trityl-*rac*-glycidol **2** (2.13 g, 80%) as yellow oil.

^{1}H-NMR (300 MHz $CDCl_3$) δ 3.20-3.28 (m, 2H, *sn*-1 CH_2), 3.5-3.61 (m, 2H, *sn*-3 CH_2), 3.99-4.02(m, 1H, *sn*-2 CH_2),4.54 (s, 2H), 7.23-7.45 (m, 20H, Ar);^{13}C-NMR (100 MHz $CDCl_3$) δ 64.7 (C *sn*-1), 70.1 (C *sn*-2) 71.7 (C sn-3) 73.5 (<u>C</u>H_2 Ar), 86.8 (<u>C</u>-Ar_3), 127.1, 127.2, 127.78, 127.81, 127.83, 127.93, 128.53, 128.69, 128.80 and 144.0 (Ar); MS calculated for $C_{29}H_{28}NaO_3^+$: 447.2. Found $[M+Na]^+$: 447.2

1-Benzyl-2-octadecyl-3-trityl-*rac*-glycerol (3)
To a solution of **2 (**1.15 g, 2.72 mmol), 1-bromooctadecane (2.26 g, 6.79 mmol), (*n*-Bu)$_4$NBr (0.087 g, 0.269 mmol) and t-BuOK (0.458 g, 4.08 mmol) were added in anhydrous toluene (10 mL). The heterogeneous reaction mixture was refluxed at 100°C for 2 h, then cooled and concentrated. The residue was dissolved in CH_2Cl_2 (30 mL), washed with H_2O (20 mL), dried with $MgSO_4$ and then concentrated. The residue was purified by silica gel column chromatography (CH_2Cl_2/petroleum ether, 33:66, v/v) providing 1-benzyl-2-octadecyl-3-trityl-*rac*-glycerol **3** (1.3 g, 68%)

^{1}H-NMR (300 MHz $CDCl_3$) δ 0.86 (t, 3H, *J*=6.7Hz, C18-CH_3), 1.23 (br s, 30H, C(3-17)-CH_2), 1.53-1.60 (m, 2H, C2-CH_2), 3.20 (s, 2 H, *sn-3* CH_2) , 3.58-3.70 (m, 5H, *sn*-1 CH_2, *sn*-2 CH, C1-CH_2), 4.50 (s, 2H, CH_2Ar), 7.26-7.53 (m, 20 H, Ar_3);^{13}C-NMR (100 MHz $CDCl_3$) δ 14.3 (C18), 22.8 (C17), 26.3 (C3), 29.5 (C15), 29.7 (C4), 29.8-29.9 (C5-C14), 30.3 (C16), 32.1 (C2), 63.6 (C1), 70.6 and 70.9 (C *sn*-1 and *sn*-3), 73.4 (<u>C</u>H_2 Ar), 78.5 (C *sn*-2), 86.7 (<u>C</u>-Ar_3), 122.0, 127.6, 127.6, 127.9, 128.4, 128.9, 138.6 and 144.3 (Ar); MS calculated for $C_{47}H_{64}NaO_3^+$: 699.5. Found $[M+Na]^+$: 699.4

1-Benzyl-2-octadecyl-*rac*-glycerol (4)
To a solution of **3** (0.8 g, 1.2 mmol), in MeOH (40 mL), camphorsulfonic acid (CSA, 55 mg, 0.24 mmol) was added. The heterogeneous reaction mixture was stirred for 24 h at room temperature and poured into a mixture of sat. aq NH_4Cl (50 mL) and water (20 mL). The aqueous phase was extracted with CH_2Cl_2 (3 x 30 mL). The combined organic solution was dried with $MgSO_4$ and then concentrated. The residue was purified by column chromatography (Pentane/Ethyl Acetate, 85:15, v/v) giving 1-benzyl-2-octadecyl-*rac*-glycerol **4** (0.44 g, 85%).

^{1}H-NMR (300 MHz $CDCl_3$) δ 0.88 (t, 3H, *J*=6.9 Hz, C18-CH_3), 1.25 (br s, 30H, C(3-17)-CH_2, 1.54-1.57(m, 2H, C2- CH_2), 2.15 (t, 1H, *J*=6.2 Hz, *sn*-3 OH), 3.44 (t, *J*=6.1 Hz, C1-CH_2), 3.56-3.69 (m, 5H, *sn*-1 CH_2, *sn*-2 CH, *sn*-3 CH_2), 4.61-4.74 (dd, 2H, *J*=11.8, 16.3 Hz, CH_2Ar), 7.30-7.36 (m, 7H, Ar); ^{13}C-NMR (100 MHz $CDCl_3$) δ 14.3 (C18), 22.8 (C17), 26.3 (C3), 29.5 (C15), 29.7 (C4), 29.8-29.9 (C5-C14,C16), 32.1 (C2), 63.2 (C1), 71.3 and 72.2 (C *sn*-1, *sn*-3 and <u>C</u>H_2 Ar), 78.0 (C *sn*-2), 127.9, 128.6 and 138.47 (Ar). MS calculated for $C_{28}H_{50}NaO_3^+$: 457.4. Found $[M+Na]^+$: 457.4

1-Benzyl-2-octadecyl-*rac*-glycero-phosphocholine (6)
To a solution of **4** (0.4 g, 0.92 mmol) in toluene (12 mL) at 0°C, a solution of trimethylamine (Et_3N 1.54 g, 13.8 mmol) and chlorophospholane ($C_2H_4ClO_3P$, 1.4 g, 13.8 mmol) were added. The mixture was stirred 24 h at room temperature and concentrated to obtain an amorphous brown compound (**5**). In a stainless steel Paar reactor, to the crude mixture dissolved in acetonitrile (30 mL) was added rapidly a solution of cold trimethylamine (TMA, 0.9 mL, 9.2 mmol). The reaction was stirred at 70°C for 18 h. After removal of the excess of TMA, the mixture was purified by column chromatography ($CHCl_3$/MeOH/H_2O, 65:35:4, v/v) and gave a white paste, 1-benzyl-2-octadecyl-*rac*-glycerol-*3*-phosphocholine **6** (0.38 g, 69%).

^{1}H-NMR (300 MHz $CDCl_3$) δ 0.89 (t, 3H, *J*=7.0 Hz, C18-CH_3), 1.24 (s, 30H, C (3-17)-CH_2), 1.50-1.53 (m, 2H, C2-CH_2), 3.53 (s, 9H N-$(CH_3)_3$), 3.57-3.68 (m, 6H,C1-CH_2, N-CH_2, *sn*-3 CH_2), 3.97 (s, 3H, *sn*-1 CH_2, *sn*-2 CH), 4.31 (s, 2H, PO-CH_2), 4.51 (dd, 2H, *J*=12.0, 14.2 Hz CH_2Ar), 7.31-7.32 (m, 5H, Ar); ^{13}C-NMR (100 MHz $CDCl_3$) δ 14.23 (C18), 22.8 (C17), 26.3 (C3), 29.5 (C15), 29.8 (C4), 29.8-30.3 (C5-C14, C16), 32.1 (C2), 54.4 ($(CH_3)_3$), 60.1 (C-N), 65.65 ($J_{P\text{-}C}$ = 5.89 Hz, C *sn*-3), 69.62 (CH_2-Ar), 70.7 (PO-OCH_2), 73.5 (C *sn*-1), 77.7 (C *sn*-2), 127.8, 127.9, 128.6, 138.5 (C Ar); ^{31}P NMR (120 MHz) δ -2.32; MS (ESI positive mode) calculated for $C_{33}H_{63}NO_6P^+$: 600.4 Found $[M+H]^+$: 600.5

2-Octadecyl-*rac*-glycero-3-phosphocholine (7)
A solution of **6** (0.530 g, 0.88 mmol) in MeOH (20 mL) with Pd/C (10%) (0.080 g) was stirred under H_2 at room temperature for 12 h. The solution was then filtered and the solvent was evaporated to give *2*-octadecyl-*rac*-glycero-*3*-phosphocholine **7** (459 mg, 99%) as a white waxy paste.

^{1}H-NMR (300 MHz $CDCl_3$) δ 0.89 (t, 3H, *J*= 6.6 Hz, C18-CH_3), 1.28-1.33 (m, 30H, C (3-17)-CH_2), 1.52-1.56 (m, 2H, C2-CH_2), 3.23 (s, 9H N-$(CH_3)_3$),3.50-3.37 (m, 5H, CH_2-N, CH *sn*-1, C1), 3.70-3.74 (m, 2H, CH_2 sn-3), 3.97-4.22 (m, 2H, PO-CH_2, CH *sn*-1), 4.43 (m, 1H, *sn*-2 CH), ^{13}C-NMR (100 MHz $CDCl_3$) δ 14.5 (C18), 23.8 (C17), 27.2 (C3), 30.5 (C15), 30.7 (C4), 30.78, 30.81, 31.2 (C5-C14, C16), 33.1 (C2), 54.60, 54.63, 54.67 ($(CH_3)_3$), 60.1 (C-N, $J_{C\text{-}p}$ = 4.40 Hz), 61.7 (C *sn*-1), 66.0 (PO-C, $J_{P\text{-}C}$ = 5.87 Hz), 67.0 (C1), 69.0 (C *sn*-3 , $J_{P\text{-}C}$=5.13), 80.4 (C *sn*-2, $J_{P\text{-}C}$ = 8.07 Hz); ^{31}P NMR (120 MHz $CDCl_3$) δ 0.24; MS (ESI positive mode) calculated for $C_{26}H_{57}NO_6P^+$: 510.4. Found $[M+H]^+$: 510.5

EOPC (8)
The compound **7** (0.1 g, 0.15 mmol) and α-eleostearic acid (0.14 g, 0.5 mmol) were dissolved in alcohol free and anhydrous $CHCl_3$ (5 mL). Freshly re-crystallized (pentane) Ppyr (0.074 g, 0.5 mmol) was dissolved with DCC (0.1 g, 0.5 mmol) in alcohol free and anhydrous $CHCl_3$

(3 mL) and stirred for 3 min. The mixture was then added dropwise to the previous reaction medium and stirred at room temperature for 24 h. The heterogeneous reaction was filtered to eliminate the DCU and purified by silica gel flash chromatography ($CHCl_3$:MeOH:H_2O, 75:25:3, v/v/v). Fractions containing the product were further purified by ion exchange chromatography using Amberlyst® 15 resin ($CHCl_3$/MeOH/H_2O, 75:25:3, v/v/v) affording EOPC (**8**, 77 mg) with 48% yield.

^{1}H-NMR (300 MHz $CDCl_3$) δ 0.85-0.91 (m, 6H, C18-CH_3 , C36-CH_3), 1.25-1.39 (m, 40H C(4-7, 16, 17, 21-35)-CH_2), 1.52-1.5 (m, 4H C(3,20)-CH_2), 2.06-2.17 (m, 4H, C(8,15)-CH_2), 2.31 (m, 2H, C2-CH_2), 3.41, (s, 9H, N-(CH_3)$_3$), 3.66-3.70 (m, 2H, CH_2-N), 4.03-4.13 (m, 4H, C (C19, *sn*-3)-CH_2), 4.25 (m, 2H, *sn*-1-CH_2), 4.52 (m, 2H, PO-CH_2), 5.36-5.39 (m, 1H, *sn*-1-CH), 5.65-5.72 (m, 2H, C(9,14)-CH), 5.94-6.20 (m, 3H, C(10, 11, 13)-CH), 6.20-6.36 (m, 1H C12-CH); ^{13}C-NMR (100 MHz $CDCl_3$) δ 14.0 (C1),14.1 (C19), 22.2 (C17), 22.7 (C35), 25.1 (C3), 26.0 (C26), 27.9 (C15), 29.5, 29.55, 29.86, 29.6, 29.96, 30.04, 31.6, 32.1, 32.7 (C4-C14, C16, C22-C25), 34.2 (C3), 54.5 ((CH_3)$_3$), 60.0 (C-N), 63.1 (PO-C, J_{C-P} = 3.67 Hz), 64.4 (C *sn-3,* J_{C-P} =5.14 Hz), 66.4 (C1), 69.3 (C *sn*-1), 71.8 (C *sn*-2 , J_{C-P} = 8.07 Hz), 126.0 (C30), 128.9 (C28), 130.7 (C31), 131.8 (C27), 133.0 (C29), 135.3 (C32), 173.9 (C19); ^{32}P-NMR (120 MHz $CDCl_3$) δ-0.92; HR-MS (ESI, positive mode) calculated for $C_{44}H_{85}NO_7P^+$: 770.6064. Found $[M+H]^+$: 770.6022

Synthesis of OEPC

1-Octadecyl-3-trityl-*rac*-glycerol (9)
Octadecanol (34 mmol, 8.12 g) in DMF (50 mL) was stirred for 30 min at 45°C until complete dissolution and cooled at room temperature. NaH (34 mmol, 1.2 g) in DMF (6 mL) was added to the previous mixture. Then, a solution of trityl-glycidol **1** (4 g, 12.6 mmol) in DMF (10 mL) was added dropwise in the reaction and stirred for 2 h 30 at 100°C. After cooling, the reaction was diluted with H_2O (40 mL) and extracted with diethyl ether (4 x 60 mL). The combined organic extract was dried with $MgSO_4$ and then concentrated. Excess of DMF was distillated off (bath temperature 100°C, 130 Pa) and the residue was purified by silica gel column chromatography (pentane/diethyl ether, 66:33, v/v) giving 1-octadecyl-3-trityl-*rac*-glycerol **9** (3.8 g, 52%)

^{1}H-NMR (300 MHz $CDCl_3$) δ 0.89 (t, 3H, *J*= 6.6 Hz, C18-CH_3) 1.25-1.29 (30H, C (3-17)-CH_2), 1.51-1.54 (m, 2H, C2-CH_2) 3.16-3.20,(m, 2H, C1-CH_2), 3.40-3.52,(m, 4H, CH_2 *sn*-1, CH_2 *sn*-3), 3.93-3.95 (m, 1H, CH *sn*-2), 7.20-7.40 (m, 15H, Ar_3); ^{13}C-NMR (100 MHz $CDCl_3$) δ 14.3 (C18), 22.8 (C17), 26.3 (C3), 29.5 (C15), 29.7 (C4), 29.8-29.9 (C5-C14), 32.1 (C2) 64.7 (C1), 70.0 (C *sn*-2), 70.6 and 70.9 (C sn-1 and *sn*-3), 86.8 (C-Ar_3), 122.2, 127.4, 127.8, 128.0-128.1, 128.8, 144.0 (C Ar); MS (ESI positive mode) calculated for $C_{40}H_{58}NaO_3^+$: 609.4. Found $[M+Na]^+$: 609.4

1-Octadecyl-2-benzyl-3-trityl-*rac*-glycerol (10)
To a solution of **9** (2 g, 3.4 mmol) in DMF (20 mL), NaH (5.1 mmol, 0.122 g) was added and stirred 15 min. After addition of benzyl bromide (BrBn, 5.1 mmol, 0.6 mL), the reaction was stirred for 24 h at room temperature. The mixture was diluted with water (30 mL) and the

aqueous phase extracted with CH_2Cl_2 (3 x 30 mL). The combined organic layers were dried with $MgSO_4$, concentrated under reduced pressure. The crude residue was purified by silica gel column chromatography (pentane/DCM, 66:33, v/v) providing 1-octadecyl-2-benzyl-3-trityl-*rac*-glycerol **10** (1.7 g, 74%) as a white paste.

^{1}H-NMR (300 MHz $CDCl_3$) δ 0.94 (t, 3H, *J*= 6.7 Hz, C18-CH_3) 1.32 (s, 30H, C (3-17)-CH_2), 1.56-1.60 (m, 2H, C2-CH_2), 3.30 (d, 2H, *J*= 4.9, CH_2 sn-1), 3.45 (t, 2H, *J*= 6.6 Hz, C1-CH_2), 3.62-3.64 (m, 2H CH_2 *sn*-3), 3.78-3.80 (m, H, CH *sn*-2), 4.71 (d, 2H, *J*= 2.2 Hz, CH_2 Ar), 7.25-7.42 (15H, CH Ar_3), 7.50-7.53 (5H, Ar);^{13}C-NMR (100 MHz $CDCl_3$) δ 14.3 (C18), 22.8 (C17), 26.3 (C3), 29.5 (C15), 29.7 (C4), 29.8-29.9 (C5-C14), 32.1 (C2) 63.8 (C1), 71.44, 71.76 (C *sn*-3 and C *sn*-1), 72.3 (C CH_2 Ar), 77.7 (C *sn*-2), 127.0, 127.6, 127.85, 127.9, 128.4, 128.5, 128.9, 139.0, 144.2 (C Ar); MS (ESI positive mode) calculated for $C_{47}H_{64}NaO_3^+$: 699.5. Found $[M+Na]^+$: 699.6

1-Octadecyl-2-benzyl-*rac*-glycerol (11)

To a solution of **10** (0.8 g, 1.2 mmol) in MeOH (40 mL), CSA (55 mg, 0.24 mmol) was added. The heterogeneous reaction mixture was stirred for 24 h at room temperature and poured into a mixture of sat. aq solution of NH_4Cl (50 mL) and water (20 mL). The aqueous phase was extracted with CH_2Cl_2 (3 x 30 mL). The combined organic solution was dried with $MgSO_4$ and then concentrated. The residue was purified by column chromatography (Pentane/Ethyl Acetate, 85:15, v/v) to give 1-octadecyl-2-benzyl-*rac*-glycerol **11** (0.47 g, 67-90%)

^{1}H-NMR (300 MHz $CDCl_3$) δ 0.88 (t, 3H, *J*= 6.7 Hz, C18-CH_3), 1.32 (s, 30H, C (3-17)-CH_2), 1.54-1.59 (m, 2H, C2-CH_2), 2.13-2.17 (m, 1H C *sn-3* OH), 3.54 (2H, td, *J*= 1.5, 6.7 Hz C1-CH_2), 3.61-3-65 (dq, 2H, *J*= 9.9, 13.8, 9.9, 14.3 Hz, CH_2 *sn*-1), 3.73-3.79 (m, 2H, CH_2 *sn*-3), 3.82-3.87 (m, 1H, CH *sn*-2), 4.65 (dd, *J*= 11.7, 23.6 Hz, 2H CH_2 Ar), 7.29-7.36 (m, 5H, Ar); ^{13}C-NMR (100 MHz $CDCl_3$) δ 14.3 (C18), 22.8 (C17), 26.3 (C3), 29.5 (C15), 29.6 (C4), 29.7-29.8 (C5-C14), 32.0 (C2) 63.2 (C1), 71.3, 72.0 (C *sn*-1 and C *sn*-3), 72.2 (C CH_2 Ar), 77.9 (C *sn*-2) 127.9, 128.6, 138.5 (C Ar); MS calculated for : $C_{28}H_{50}NaO_3^+$ 457.4. Found $[M+Na]^+$: 457.4

1-Octadecyl-2-benzyl-*rac*-glycero-3-phosphocholine (13)

To a solution of **11** (0.8 g, 1.8 mmol) in toluene (12 mL) a solution of triethylamine (0.46 g, 4.6 mmol) and chlorophospholane ($C_2H_4ClO_3P$, 0.65 g, 4.6 mmol) were added at 0°C. The mixture was stirred for 24 h at room temperature and concentrated to obtain an amorphous brown compound (**12**). In a stainless steel Paar reactor, to the crude mixture dissolved in acetonitrile (30 mL) was added rapidly a solution of cold trimethylamine (TMA, 1.68 mL, 18 mmol). The reaction was stirred at 70°C for 18h. The excess of TMA was removed, the mixture was purified by column chromatography ($CHCl_3$/MeOH/H_2O, 65:35:4, v/v/v) and gave a white paste 1-octadecyl-2-benzyl-*rac*-glycero-3-phosphocholine **13** (0.88 g, 98%).

^{1}H-NMR (300 MHz $CDCl_3$) δ 0.87 (t, 3H, *J*=6.9 Hz, C18-CH_3), 1.23 (s, 30H, C (3-17)-CH_2), 1.51 (t, 2H, *J*= 6.1 Hz, C2-CH_2), 3.08 (s, 9H, N-$(CH_3)_3$), 3.37 (t, 2H, *J*=6.6Hz, C1-CH_2), 3.49-3.52 (m, 3H, CH_2-N, CH *sn*-1), 3.73 (s, H, *sn*-1 CH), 3.89-3.91(m, 2H, CH_2 *sn*-3), 4.12-4.15 (m, 3H, PO-CH_2, CH *sn*-2), 7.21-7.36 (m, 5H, C-Ar); ^{13}C-NMR (100 MHz $CDCl_3$) δ 14.3 (C18), 22.8 (C17), 26.3 (C3), 29.5 (C15), 29.79 (C4), 29.81-30.1 (C5-C14,C16), 32.1 (C2),

54.3 ($(CH_3)_3$), 60.0 (J_{C-P} = 4.4 Hz, C CH_2-N), 65.6 (J_{C-P} =5.9 Hz, PO-CH_2), 65.9 (J_{C-P} =6.6 Hz, C *sn*-3), 69.6 (C1), 70.7 (C *sn*-1), 73.5 (CH_2-Ar), 77.7 (C *sn*-2), 127.8, 128.5 (C Ar); ^{31}P NMR (120 MHz) δ -0.93; MS (ESI positive mode) calculated for $C_{33}H_{63}NO_6P^+$: 600.4. Found $[M+H]^+$: 600.5

1-Octadecyl–*rac*-glycero-3-phosphocholine (14)

A solution of **13** (230 mg, 0.38 mmol) in MeOH (10 mL) with Pd/C (10%) (35 mg) was stirring for 12 h at room temperature under H_2 atmosphere. The solution was then filtered on Celite and concentrated. 1-Octadecyl-*rac*-glycero-*3*-phosphocholine **14** (459 mg, 99%) was obtained as a white waxy paste.

^{1}H-NMR (300 MHz MeOD) δ 0.90 (t, 3H, *J*=6.9 Hz, C18-CH_3), 1.29 (s, 30H, C (3-17)-CH_2), 1.51 (t, 2H, *J*= 6.8 Hz C2-CH_2), 3.23 (s, 9H, N-$(CH_3)_3$), 3.46-3.48 (m, 4H C1, CH_2-N), 3.65-3.67 (m, 2H, C *sn*-1), 3.88-3.95 (C *sn*-1 and *sn*-2), 4-30-4.31 (m, 2H, PO-CH_2); ^{13}C-NMR (100 MHz $CDCl_3$) δ 14.5 (C18), 23.7 (C17), 27.2 (C3), 30.4 (C15), 30.66 (C4), 30.7 (C5-C14,C16), 33.1 (C2), 54.7 ($(CH_3)_3$), 60.4 (J_{C-P} = 5.14 Hz, C CH_2-N), 65.9 (J_{C-P} =2.2 Hz, PO-CH_2), 67.4 (C1), 68.5 (J_{C-P} =5.9 Hz, C *sn*-1), 70.9 (C *sn*-2), 72.7 (C *sn*-2); ^{31}P NMR (120 MHz) δ -0.8; MS (ESI positive mode) calculated for $C_{26}H_{57}NO_6P^+$:510. Found $[M+H]^+$: 510.5

OEPC (15)

The compound **14** (100 mg, 0.075 mmol) and α-eleostearic acid (140 mg, 0.5 mmol) were dissolved in alcohol free and anhydrous $CHCl_3$ (5 mL). Freshly re-crystallized (pentane) Ppyr (74 mg, 0.5 mmol) was dissolved with DCC (100 mg, 0.5 mmol) in alcohol free and anhydrous $CHCl_3$ (3 mL) and stirred for 3 min. The mixture was then added dropwise to the previous reaction medium and stirred at room temperature for 24 h. The heterogeneous reaction was purified by flash chromatography on silica gel ($CHCl_3$/MeOH/H_2O, 75:25:3, v/v/v). Fractions containing the product were further purified by ion exchange chromatography using Amberlyste® 15 resin ($CHCl_3$/MeOH/H_2O, 75:25:3, v/v/v). EOPC **15**, (20 mg) was obtained with 25% yield.

^{1}H-NMR (400 MHz $CDCl_3$) δ 0.87-0.91 (m, 6H, C(18, 36)-CH_3), 1.25-1.37 (m, 40H C (22-25, 34, 35, 3-17)-CH_2), 1.52-1.5 (m, 4H C(2, 21)-CH_2), 2.06-2.17 (m, 4H, C(26,33)-CH_2), 2.31 (m, 2H, C20-CH_2), 3.38, (s, 9H, N-$(CH_3)_3$), 3.40-3-49 (m, 2H, C1), 3.93-4.06 (m, 5H, CH_2-N, CH_2 *sn*-1, CH *sn*-3), 4.23-4.25 (m, H, CH *sn*-3), 4.43 (s, 2H, PO-CH_2), 5.12-5.15 (1H,CH *sn*-2), 5.34-5.39 (m, H, C9-CH), 5.65-5.71 (m, H, C14-CH), 5.95-6.18 (m, 3H, (C10, C11, C13)-CH), 6.33-6.39 (m, H, C12-CH); ^{13}C-NMR (100 MHz $CDCl_3$) δ 14.1 (C1),14.3 (C19), 22.4 (C17), 22.8 (C35), 25.1 (C3), 26.2 (C26), 28.0 (C15), 29.2, 29.3, 29.4, 29.5, 29.72, 29.78, 29.82, 29.87, 29.90,(C4-C14, C16, C22-C25), 31.6,(C3), 32.6 (C3), 54.6 ($(CH_3)_3$), 60.3, (C-N), 60.4 (PO-C, J_{C-P} = 5.4 Hz), 66.2 (C *sn-3*, J_{C-P} =8.07), 69.1 (C1), 71.9 (C *sn*-1), 77.4 (C *sn*-2), 126.1 (C30), 128.9 (C28), 130.7 (C31), 132.0 (C27), 133.0 (C29), 135.4 (C32), 173.5 (C19); ^{31}P NMR (120 MHz) δ -2.25; HR-MS (ESI, positive mode) calculated for $C_{44}H_{85}NO_7P^+$: 770.6064, Found H^+ 770.6029

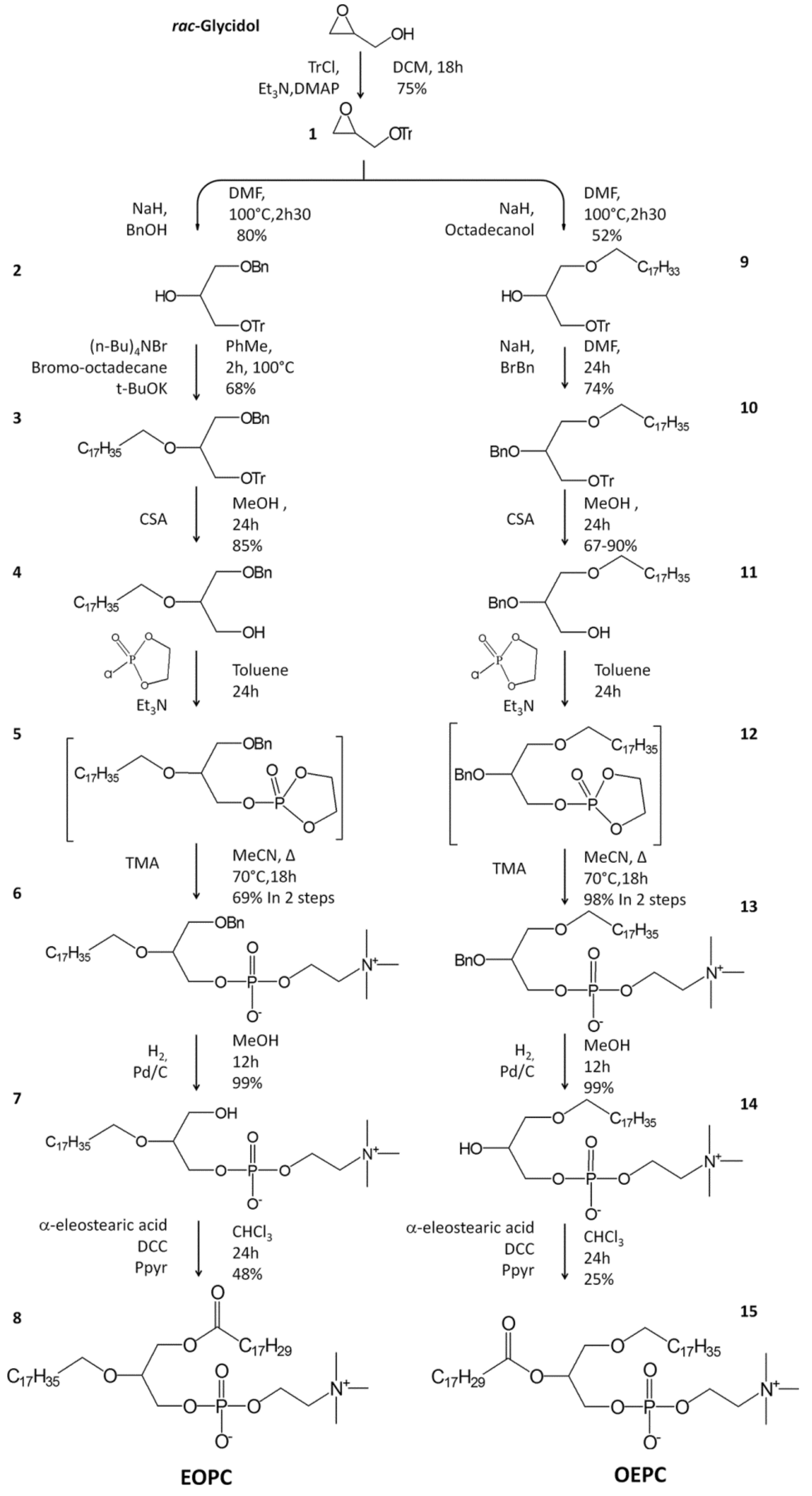

Figure S1: Synthesis of EOPC and OEPC in eight steps from *rac*-glycidol.

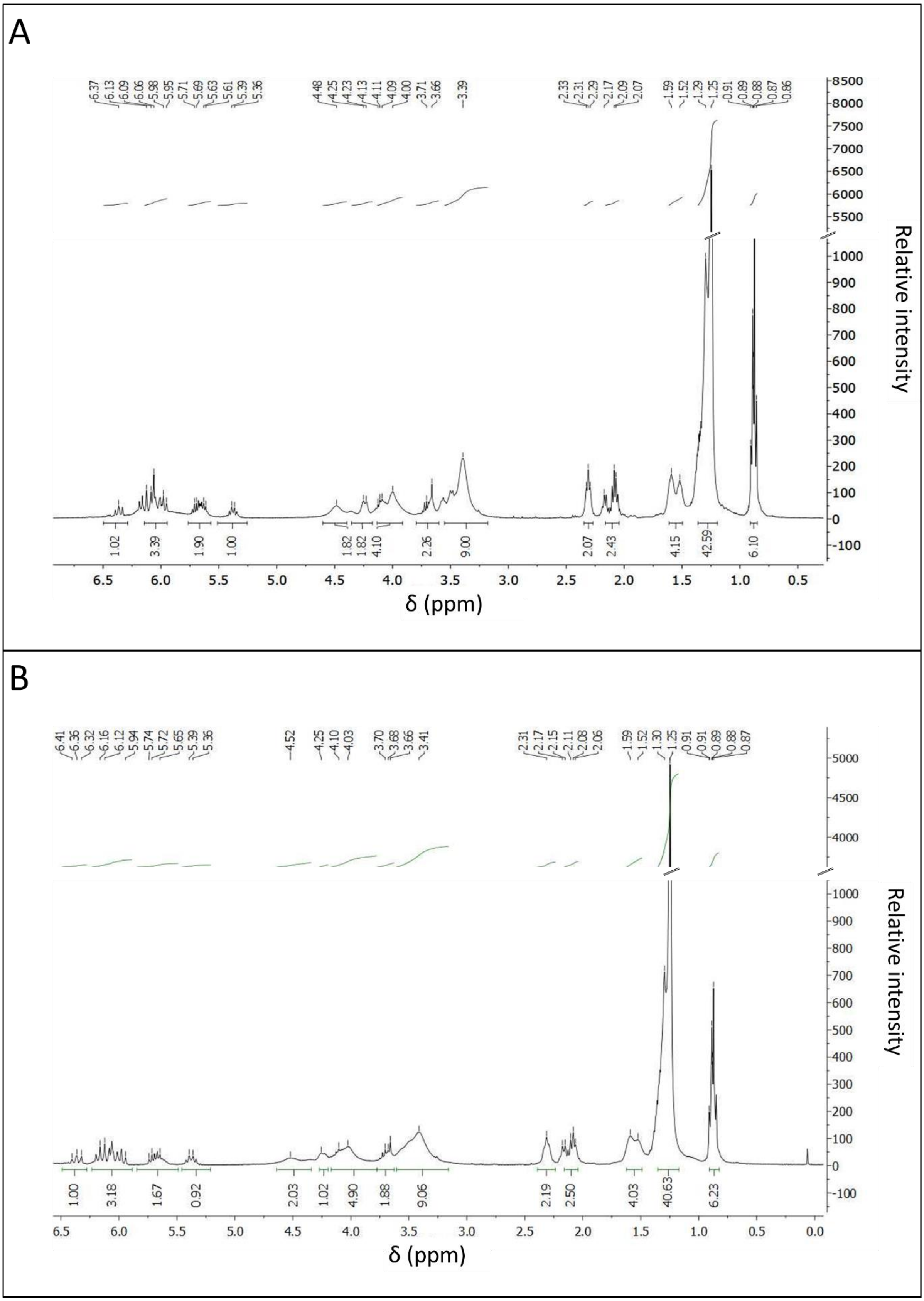

Figure S2: ^{1}H NMR (400 MHz) spectrum of pure EOPC (**A**) and pure OEPC (**B**) in deuterated chloroform. Chemical shifts are given in δ-values in ppm downfield from the chloroform ($\delta CHCl_3$ = 7.26).

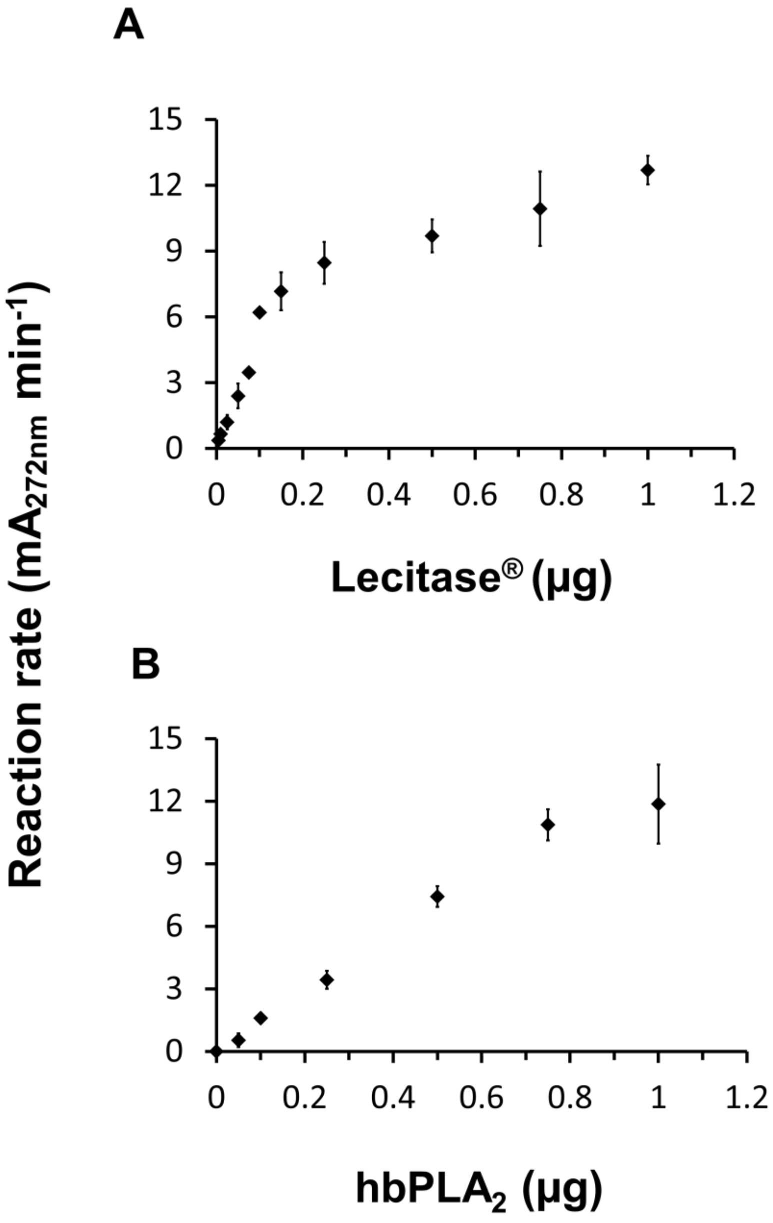

Figure S3: Effects of the amount of enzyme on the steady state reaction rate. Effects of varying amounts of enzyme on the steady state reaction rate using coated EOPC (A) or coated OEPC (B). Variable amounts of Lecitase® (A) or hbPLA$_2$ (B) were injected into the microplate well, containing the coated PC, in 200 µL of standard buffer. The increase in the absorbance at 272 nm was recorded for 20-40 min after the enzyme injection, and the initial velocity ($mA_{272nm}.min^{-1}$) was used for reaction rate determination. Results are given as means ± SD for three independent experiments.

REFERENCES

1. White, J. D., C. M. Lincoln, J. Yang, W. H. Martin, and D. B. Chan. 2008. Total synthesis of solandelactones A, B, E, and F exploiting a tandem Petasis-Claisen lactonization strategy. *J Org Chem* **73**: 4139-4150.
2. Hocek, M., M. Masojídková, A. Holý, G. Andrei, R. Snoeck, J. Balzarini, and E. De Clercq. 1996. Synthesis and Antiviral Activity of Acyclic Nucleotide Analogues Derived from 6-(Aminomethyl)purines and Purine-6-carboxamidines. *Collection of Czechoslovak Chemical Communications* **61**: 1525-1537.
